# The first *in vivo* multiparametric comparison of different radiation exposure biomarkers in human blood

**DOI:** 10.1371/journal.pone.0193412

**Published:** 2018-02-23

**Authors:** Ales Tichy, Sylwia Kabacik, Grainne O’Brien, Jaroslav Pejchal, Zuzana Sinkorova, Adela Kmochova, Igor Sirak, Andrea Malkova, Caterina Gomila Beltran, Juan Ramon Gonzalez, Jakub Grepl, Matthaeus Majewski, Elizabeth Ainsbury, Lenka Zarybnicka, Jana Vachelova, Alzbeta Zavrelova, Marie Davidkova, Marketa Markova Stastna, Michael Abend, Eileen Pernot, Elisabeth Cardis, Christophe Badie

**Affiliations:** 1 Department of Radiobiology, Faculty of Military Health Sciences, Hradec Králové, University of Defence in Brno, Hradec Králové, Czech Republic; 2 Biomedical Research Centre, University Hospital, Hradec Králové, Czech Republic; 3 Cancer Mechanisms and Biomarkers group, Radiation Effects Department, Centre for Radiation, Chemical and Environmental Hazards, Public Health of England, Didcot, United Kingdom; 4 Department of Toxicology, Faculty of Military Health Sciences, Hradec Králové, University of Defence in Brno, Czech Republic; 5 Department of Oncology and Radiotherapy and 4th Department of Internal Medicine - Hematology, University Hospital, Hradec Králové, Czech Republic; 6 Department of Hygiene and Preventive Medicine, Faculty of Medicine in Hradec Králové, Charles University in Prague, Hradec Králové, Czech Republic; 7 Institute for Global Health, Barcelona, Spain; 8 Bundeswehr Institute of Radiobiology, Munich, Germany; 9 Department of Radiation Dosimetry, Nuclear Physics Institute of the Czech Academy of Sciences, Prague, Czech Republic; 10 Institute for Hematology and Blood Transfusion, Hospital Na Bulovce, Prague, Czech Republic; Northwestern University Feinberg School of Medicine, UNITED STATES

## Abstract

The increasing risk of acute large-scale radiological/nuclear exposures of population underlines the necessity of developing new, rapid and high throughput biodosimetric tools for estimation of received dose and initial triage. We aimed to compare the induction and persistence of different radiation exposure biomarkers in human peripheral blood *in vivo*. Blood samples of patients with indicated radiotherapy (RT) undergoing partial body irradiation (PBI) were obtained soon before the first treatment and then after 24 h, 48 h, and 5 weeks; i.e. after 1, 2, and 25 fractionated RT procedures. We collected circulating peripheral blood from ten patients with tumor of endometrium (1.8 Gy per fraction) and eight patients with tumor of head and neck (2.0–2.121 Gy per fraction). Incidence of dicentrics and micronuclei was monitored as well as determination of apoptosis and the transcription level of selected radiation-responsive genes. Since mitochondrial DNA (mtDNA) has been reported to be a potential indicator of radiation damage *in vitro*, we also assessed mtDNA content and deletions by novel multiplex quantitative PCR. Cytogenetic data confirmed linear dose-dependent increase in dicentrics (p < 0.01) and micronuclei (p < 0.001) in peripheral blood mononuclear cells after PBI. Significant up-regulations of five previously identified transcriptional biomarkers of radiation exposure (*PHPT1*, *CCNG1*, *CDKN1A*, *GADD45*, and *SESN1*) were also found (p < 0.01). No statistical change in mtDNA deletion levels was detected; however, our data indicate that the total mtDNA content decreased with increasing number of RT fractions. Interestingly, the number of micronuclei appears to correlate with late radiation toxicity (r^2^ = 0.9025) in endometrial patients suggesting the possibility of predicting the severity of RT-related toxicity by monitoring this parameter. Overall, these data represent, to our best knowledge, the first study providing a multiparametric comparison of radiation biomarkers in human blood *in vivo*, which have potential for improving biological dosimetry.

## Introduction

There is an identified need to improve simple and efficient biodosimetric tools that could be used for triage purposes in public situations such as the screening of potential victims of a nuclear accident, an act of radiologic terrorism or military conflict [1,2]. Such tools would also be of a great use to refine radiation exposure classification in retrospective or large-scale molecular epidemiology studies where biomarkers of exposure are currently lacking.

In order to assess the level of exposure of individuals, the development of a high-throughput assay that could process large numbers of samples in a short period is needed. The conventional methods such as dicentric chromosomes (DC) or micronuclei (MN) assessment are providing reliable data [3], nevertheless, they are also technically demanding and time consuming (2–3 days), which is far from optimal for situations with the risk of exposure to ionizing radiation (IR) when thousands of irradiated persons are expected. In the situations where speed and throughput are more important than accuracy, it would be extremely helpful to develop other effective countermeasures for biodosimetric triage. Thus, rapid molecular assays have the potential to become useful triage tools.

Breakage of cellular DNA following IR exposure occurs in humans within two interdependent genomes of nuclear and extra-nuclear DNA. Several nuclear DNA-based approaches have been reported to possess the potential to provide *ex-post* information regarding exposure to IR such as scoring gamma-H2AX [3] or monitoring a shift in transcriptional expression of radiation-responsive genes [4–7]. Manning *et al*. studied high and low dose responses of transcriptional biomarkers in *ex vivo* X-irradiated human blood and found *FDXR*, *DDB2*, *CCNG1* genes to be suitable transcriptional radiation exposure markers [5]. It has also been recently shown that the *ex vivo* irradiated response produces similar dose estimates to *in vivo* irradiated patient samples [8].

Apart from promising gene expression biomarkers, we aimed to investigate the extra-nuclear mitochondrial DNA (mtDNA), which was driven by the fact that it is lacking histone protection and chromatin structure, and its genetic information is more prone to DNA damage than nuclear DNA. Indeed, mtDNA damage was reported to be more extensive and transient than nuclear one [9] making it a potentially attractive source of radiation biomarker. Using several human cell lines, Prithivirajsingh *et al*. reported significant levels of mtDNA deletions 72 h following irradiation by doses ranging from 2 to 20 Gy in all of tested cell lines with lower response in tumor cell lines [10]. However, they reported no consistent dose–response relationship.

*In vivo*, evidence was found that radiotherapy (RT) is associated with an increase in mitochondrial genome mutation rate [11]. Encouragingly, Wen *et al*. have reported an increase in mtDNA deletions in peripheral lymphocytes of acute lymphoblastic leukemia patients undergoing total body irradiation (TBI) therapy [12]. Philips *et al*. recently reported a sensitive multiplex real-time polymerase chain reaction (MQRT-PCR) assay for simultaneous quantification of mitochondrial DNA copy number and deletion ratio for quantification of mtDNA site in the minor arc (mtMin) where large deletions are rare and another mtDNA site in the major arc (mtMaj) where large deletions are common. In fact, approximately 84% of published deletions span the mtMaj target [13]. Thus, using this sensitive assay, we hypothesized that mtDNA could be a useful indicator of radiation exposure *in vivo* and we investigated whether IR induces mtDNA deletions using a specifically designed MQRT-PCR.

Many radiobiological studies are currently focused on identification of novel biomarkers and their validation [14]. The purpose of this study was to evaluate some new emerging biomarkers and compare them with the established ones. This study was carried out in circulating peripheral blood mononuclear cells (PBMC) of oncological patients with indicated RT undergoing partial body irradiation (PBI) of a large body volume; i.e. ten patients with tumor of endometrium and eight patients with tumor of head and neck (Table 1). MQRT-PCR provided simultaneous assessment of mtDNA copy number and proportion of mitochondria genomes with common deletions. Moreover, we compared the obtained data with analysis of DC and MN in the same samples. We also provided comparison with monitoring of transcriptional expression of *in vivo* validated radiation-responsive genes. Additionally, we determined changes in apoptotic populations in lymphocytes, lymphocytes and monocytes (PBMC) and granulocytes.

**Table 1:** The table of endometrial and head and neck patients analysed in this study.

pateint code	sex	age	diagnosis	tumor grade	5 week interval sampled	body blood volume [dm^3^]	volume (= mass) [dm^3^ = kg]	irradiated blood volume [dm3]	mean dose [%]	dose per fraction [Gy]	acute toxicity	late toxicity	micronuclei (control)	1 RT	2 RT	25 RT
E1	F	75	C542	2	yes	4,8	20,0	1,1	42,0	0,17	2	1	29	42	51	111
E2	F	64	C542	2	yes	4,9	26,6	1,4	40,3	0,21	1	0	28	29	45	116
E3	F	79	C542	1	yes	4,3	19,4	1,1	42,4	0,19	2	1	29	30	35	198
E4	F	65	C542	1	yes	3,9	18,0	1,0	44,5	0,20	2	1	26	25	30	132
E5	F	71	C542	3	yes	4,4	21,2	1,2	39,6	0,19	1	1	24	28	38	105
E6	F	57	C542	2	yes	4,1	17,2	0,9	41,4	0,16	2	1	19	27	31	132
E7	F	69	C542	2	yes	3,6	17,0	0,9	47,0	0,22	3	4	35	34	40	206
E8	F	78	C541	2	yes	4,9	22,7	1,2	38,0	0,16	2	1	23	31	38	165
E9	F	74	C548	1	yes	3,8	17,6	0,9	38,6	0,17	1	3	28	35	34	199
E10	F	74	C541	2	yes	5,2	24,5	1,3	42,5	0,19	2	0	33	35	39	127
Average		**70,6**				**4,4**	**20,4**	**1,1**	**41,6**	**0,188**			**dicentrics**	**1 RT**	**2 RT**	**25 RT**
N1	F	52	C800	2	yes	4,4	7,1	0,4	43,9	0,08	1	1	—	—	—	—
N2	M	57	C080	3	yes	6,2	11,7	0,7	46,5	0,11	1	†	2	16	12	16
N3	M	81	C443	3	yes	5,4	9,6	0,6	38,5	0,09	1	†	0	4	10	14
N4	M	79	C099	2	yes	5,0	6,7	0,4	42,5	0,07	2	2	0	8	18	34
N5	M	55	C01	2	yes	4,1	8,2	0,5	40,3	0,11	2	3	2	8	20	36
N7	M	52	C321	3	no	6,2	10,9	0,7	41,2	0,09	2	2	4	16	22	—
N8	M	66	C138	2	no	4,7	7,3	0,5	40,1	0,08	1	1	6	16	18	—
N9	M	66	C328	3	no	4,6	7,5	0,5	41,1	0,09	1	1	8	14	18	—
Average		**63,5**				**5,1**	**8,6**	**0,5**	**41,8**	**0,092**						

An overview of data used for physical dosimetry analyses is given along with radiotoxicity evaluation and cytogenetic data. Diagnosis is provided according to International Classification of Diseases. Number of micronuclei or dicentrics before RT (control), or after 24 hours (1 RT), 48 hours (2 RT), and 5 weeks (25 RT) is shown. F = female, M = male.

^†^Early death of a patient.

This multiparametric approach allowed us to monitor simultaneously several biomarkers of radiation exposure, which is, to our best knowledge, the first attempt to provide such a comparison in human blood samples irradiated *in vivo*. Besides, the cumulative doses used in this study reach high levels, therefore we attempted to use particular biomarkers to monitor radiotherapy responses as well.

## Material and methods

### Blood samples, physical doses delivered to the patients and radiation toxicity grading

Ten endometrial and eight head and neck oncological patients with indicated PBI without previous (or concomitant) radio- and chemo-therapy were enrolled in this study (Table 1). Both patients’ subgroups were treated for the same tumor localisation in order to prevent the variability usually observed among patients treated with RT and to allow the corresponding roles of the size of irradiation field and of the dose rate.

The informed consent was obtained from each individual and local Ethical Committee of University Hospital in Hradec Kralove (Czech Republic) approved experimentation with human subjects according to The Code of Ethics of the World Medical Association—Declaration of Helsinki (approval no: 201401-S15P). Ten healthy donors of corresponding age and sex were used as a control group.

**Fig 1:**
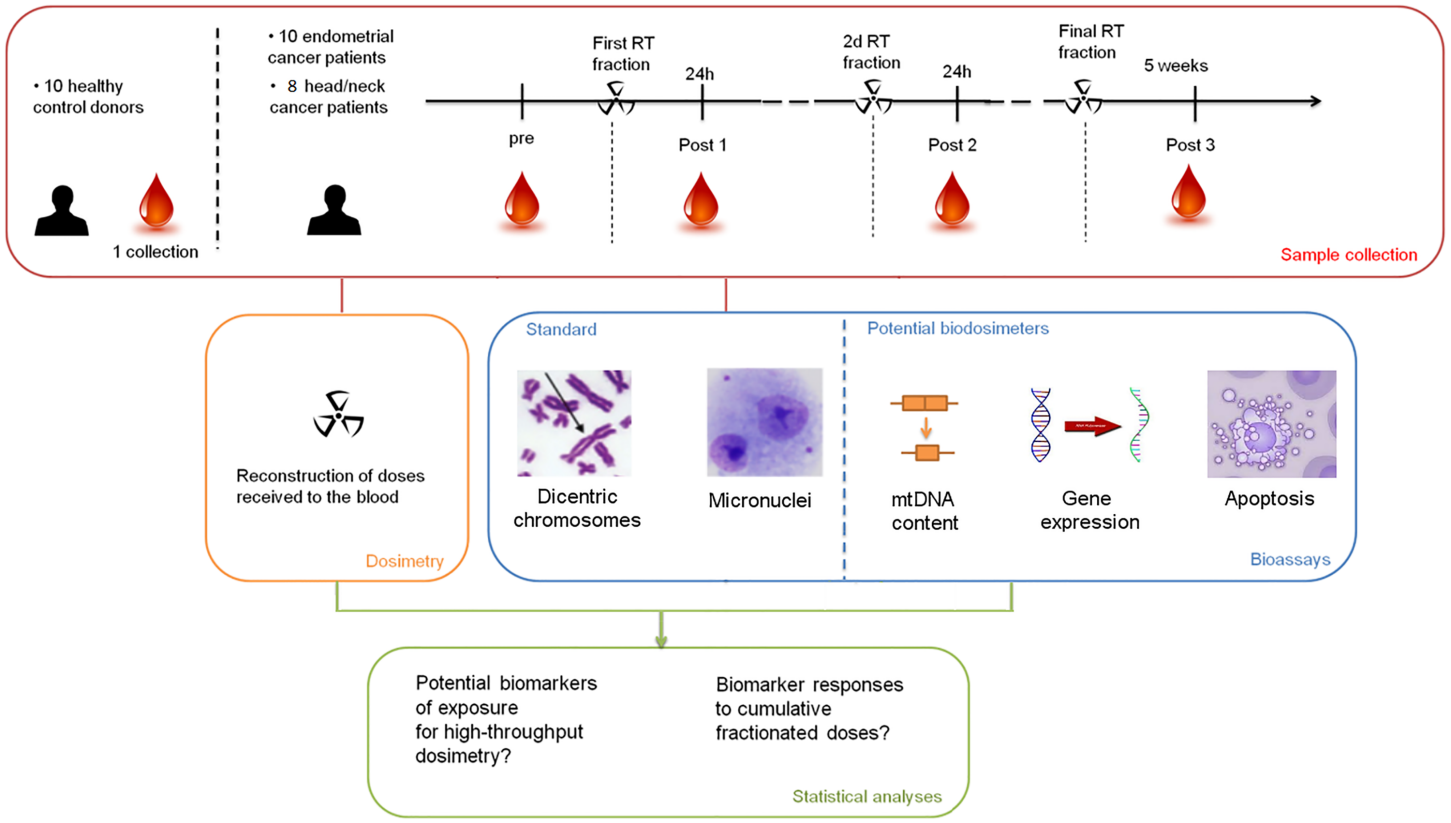
Experimental design. A scheme of blood sampling and subsequent analyses performed.

Peripheral blood samples were collected into Li-heparinized tubes (Vacuette, Mundelein, IL, USA) soon before the first treatment, which served as a control sample for determination of basal level prior to the radiation and then after 24 h, 48 h, and 5 weeks; i.e. after 1, 2, and 25 fractionated RT procedures (Fig 1). The samples were placed in the fridge and processed for subsequent cytogenetic analysis within 2 h after collection. The samples for flow-cytometry were processed directly after sampling. The samples for gene expression analysis were collected using PAXgene Blood RNA tubes according to the manufacturer’s instructions (Qiagen, PreAnalytiX GmbH, Hilden, Germany).

In the group of endometrial tumor patients, the prescribed dose was 45 Gy within 25 RT fractions which were applied during 35 days (5 weeks) using LINAC with a dose rate of 300 MU/min (Varian Medical Systems Inc., Palo Alto, CA, USA) with the treatment planning system Eclipse (Varian Medical Systems). The single dose per fraction was 1.8 Gy. The average dose delivered to the blood was calculated as 0.19 Gy per fraction. In the group of head and neck tumor patients, the prescribed dose was between 50 and 70 Gy within 25 to 33 fractions applied during 35 to 45 days. The single dose per fraction was 2.0 or 2.121 Gy (Table 1). The average dose delivered to the blood was calculated as 0.08 Gy per fraction. No shielding was used for both groups of patients.

Radiation toxicity of treatment was recorded for each patient (Table 1). Acute toxicity grading was evaluated as the worst grade of toxicity recorded during the treatment or up to 3 months after the end of the treatment—CTCAE v. 4.0 grading system was used. The full definition of the grading system can be found at the RTOG website [15]. Late toxicity grading was evaluated as the worst grade of symptoms, persisting more than 3 months after the end of the treatment

### Chromosomal aberrations

For this assay, 0.8 ml of heparinized whole blood was utilized. The level of chromosomal aberrations in peripheral lymphocytes was evaluated by a standardized method based on microscopic analysis of lymphocytic chromosomes undergoing mitotic metaphase [16]. Briefly, whole blood was cultivated and peripheral lymphocytes were stimulated by phytohemagglutinin (Gibco^®^, Waltham, MA, USA), treated with colcemid solution (Serva, Heidelberg, Germany), harvested and stained with Giemsa (Dr. Kulich Pharma, Hradec Kralove, Czech Republic). In each blood sample, 100 of mitotic sets were evaluated at 100-fold original magnification and immersion oil. We determined number of DC and structurally aberrant cells. The protocol is available at https://dx.doi.org/10.17504/protocols.io.mjgc4jw.

### Micronuclei assay

For MN assay, 4 ml of blood was used. The lymphocytes were separated from the whole blood using Histopaque gradient liquid-1077 (Sigma) according to the manufacturer´s instructions. After the separation, the cells were cultivated, stimulated with phytohemagglutinin, treated with cytochalasin B, and stained with Giemsa (all from Sigma) as we previously described in Kmochova *et al*. [17]. Stained samples were evaluated using a BX-51 microscope (Olympus, Prague, Czech Republic) at 40-fold original magnification. For scoring, criteria presented by Fenech *et al*. were used [18]. A total of 1000 binucleated cells was evaluated for the frequency of MN. The protocol is available on-line at https://dx.doi.org/10.17504/protocols.io.mr6c59e.

### Flow cytometry

In apoptosis assessment experiment, 300 μl of heparin (Zentiva, Czech Republic) were added to peripheral blood obtained from 10 healthy volunteers and radiation exposed patients. The peripheral blood was kept at the room temperature and lysed by the EasyLyse, erythocyte lysing reagent (DAKO, Glostrup, Denmark) according to manufacturer´s instructions. The cell suspension density was set to 5 x 10^6^ cells/ml in diluted Binding buffer (1:10). Lymphocytes, lymphocytes and monocytes (PBMC), and granulocytes were stained with monoclonal antibody Annexin V-FITC (BD Biosciences Pharmingen, San Jose, CA, USA) for 10 min on ice. Five μl of propidium iodide (250 μg/ml) were added after one washing step in an ice cold Washing and staining buffer. Data were acquired on CyAn ADP flow cytometer (Beckman Coulter, Fullerton, CA, USA) and analysis was performed using the Summit v4.3 software (Beckman Coulter), where lymphocytes, PBMC, and granulocytes were divided based on forward scatter / side scatter characteristics and cell death was assessed in lymphocytes, PBMC, and granulocytes populations. The protocol is available on-line at https://dx.doi.org/10.17504/protocols.io.mjhc4j6.

### mtDNA deletions measurement

Genomic DNA from 100 μl of blood was extracted using DNeasy Blood & Tissue Kit according to the manufacturer's protocol (Qiagen, Hilden, Germany). The measurement of the mtDNA deletions was done according to protocol published by Phillips et al with minor modifications. Briefly, 1 ng of DNA from patients was used in the reaction. All reactions were run in triplicate using PerfeCTa^®^ MultiPlex qPCR SuperMix (Quanta Biosciences, Inc. Gaithersburg, MD, USA) with primer and probe sets for target genes at 300 nM concentration each. FAM, HEX and Texas Red were used as fluorochrome reporters for the hydrolysis probes analysed in multiplexed reaction. Cycling parameters were 10 min at 95°C, then 40 cycles of 10 s at 95°C and 60s at 60°C. Data were collected and analysed by Rotor-Gene Q Series Software. Ct values were converted to ng using standard curves obtained by serial dilution of a control DNA sample. The linear dynamic range of the standard curves ranging from 17 ng to 66 pg gave PCR efficiencies between 93% and 103% for each assay with R2 > 0.998. Values for mitochondrial mtMaj and mtMin assays were normalized to genomic beta-2-microglobulin (B2M) internal control. The protocol including the sequences of primers and Locked Nucleic Acid (LNA) TaqMan probes is available at https://dx.doi.org/10.17504/protocols.io.mpqc5mw.

### RNA Extraction

Blood samples were collected from the radiotherapy treated cancer patients in PAXGene tubes according to the manufacturer's protocol (Qiagen, PreAnalytiX GmbH, Hilden, Germany). The tubes were kept at RT for 2 hr before being frozen at -20°C. RNA was extracted from the samples using the PAXGene Blood miRNA Kit (Qiagen, PreAnalytiX GmbH, Hilden, Germany) according to the manufacturer’s protocol. RNA quantity was assessed by Nanodrop ND2000 (Nanodrop, Wilmington, USA), and RNA quality was assessed by RIN values produced by Tapestation 2200 (Agilent Technologies, CA, USA).

### Gene expression—MQRT-PCR

Reverse transcriptase reactions were performed using High Capacity cDNA Reverse transcription kit (Applied Biosystems, FosterCity, CA, USA) according to the manufacturer ‘ s protocol with 350 ng of total RNA. Multiplex quantitative RT-PCR Real-time PCR was performed using Rotor-Gene Q (Qiagen, Hilden, Germany). All reactions were run in triplicate using PerfeCTa^®^ MultiPlex qPCR SuperMix (Quanta Biosciences, Inc. Gaithersburg, MD, USA) with primer and probe sets for target genes at 300 nM concentration each. 3 ′ 6-Carboxyfluorescein (FAM), 6-Hexachlorofluorescein (HEX), Texas Red and CY5 (all from Eurogentec Ltd, Fawley, Hampshire, UK) were used as fluorochrome reporters for the hydrolysis probes analysed in multiplexed reactions with between 2 and 4 genes per run including the control. Cycling parameters were 2 min at 95°C, then 45 cycles of 10 s at 95°C and 60 s at 60°C. Data were collected and analyzed by Rotor-Gene Q Series Software. Gene target Ct (cycle threshold) values were normalized to a Hypoxanthine-Guanine phosphoribosyltransferase 1 (HPRT1) internal control. Primer and probe designs can be found in our previous work, Kabacik *et al*. [4]. Ct values were converted to transcript quantity using standard curves obtained by serial dilution of PCR-amplified DNA fragments of each gene. The linear dynamic range of the standard curves covering six orders of magnitude (serial dilution from 3.2 x 10^−4^ to 8.2 x a10^-10^) gave PCR efficiencies between 93% and 103% for each gene with R2 > 0.998. Relative gene expression levels after irradiation were determined relative to unexposed controls.

The protocol including primers/probes sequences is available on-line at https://dx.doi.org/10.17504/protocols.io.mqic5ue.

### Physical dosimetry calculations

To determine the *Mean dose* of the irradiated blood sample of patients undergoing RT, the treatment planning system Eclipse was used, i.e. a software frequently used in external beam planning. This system uses the Anisotropic Analytical Algorithm to compute 3D dose distribution in patient’s volume. The software displays the geometry of the patient acquired by computed tomography and computed dose distribution. From these data *Irradiated Volume* (IV) defined as a volume surrounded by five percent isodose and *Mean dose* in this volume (D_mean_) were determined for each patient. Five percent isodose is the surface that connects points in 3D dose distribution where the dose is equal to 5% of the prescribed dose. It was used in order to increase consistency of the data, since the CT scans covered different body parts. Thus, for the calculation purposes, doses lower than 5% of the prescribed dose were not taken into account. In the following relation, we assume that the blood in the patient's body is irradiated homogeneously, the blood is stored in the human body homogeneously and 1 dm^3^ of human body weighs approximately 1 kg. *Mean dose* in the patient's blood (MBD) was calculated according to the relation:

$$MBD=\frac{D_{mean}.\;IBV}{BBV}$$

$$MBD=\frac{D_{mean}.\;IV}{V}$$

MBD—mean blood dose; D_mean_—irradiated volume mean dose; IBV—irradiated blood volume; IV—irradiated volume; BBV—body blood volume; V—total patient volume (approximated to patient’s weight).

### Statistical analysis

Statistical analysis of the biological data was performed using Minitab 17 (Minitab Ltd., Coventry, UK) and SPSS Statistics v24 (IBM, Armonk, NY, USA). Data points represent the mean ± SEM. All data were tested for normal distribution using Shapiro-Wilk test.

For analysis of MN data, Mann–Whitney test was used. Changes were considered statistically significant with p < 0.001.

For analysis of DC data, ANOVA was used for normal variables and Kruskal-Wallis test was used for non-normal variables. Next, univariate General Linear Model based on Hermite distribution was adjusted for the count of DC (response variable) by number of RT fractions (explanatory variable). Changes were considered statistically significant with p < 0.01.

For analysis of QRT-PCR data, paired t-test was applied. When normality test failed Mann-Whitney rank sum test was used with p < 0.01 or p < 0.005, respectively.

The dose estimates were performed as described in Abend et al. [7].

## Results

### The number of chromosomal changes in PBMCs of head and neck patients increased with number of RT fractions

**Fig 2:**
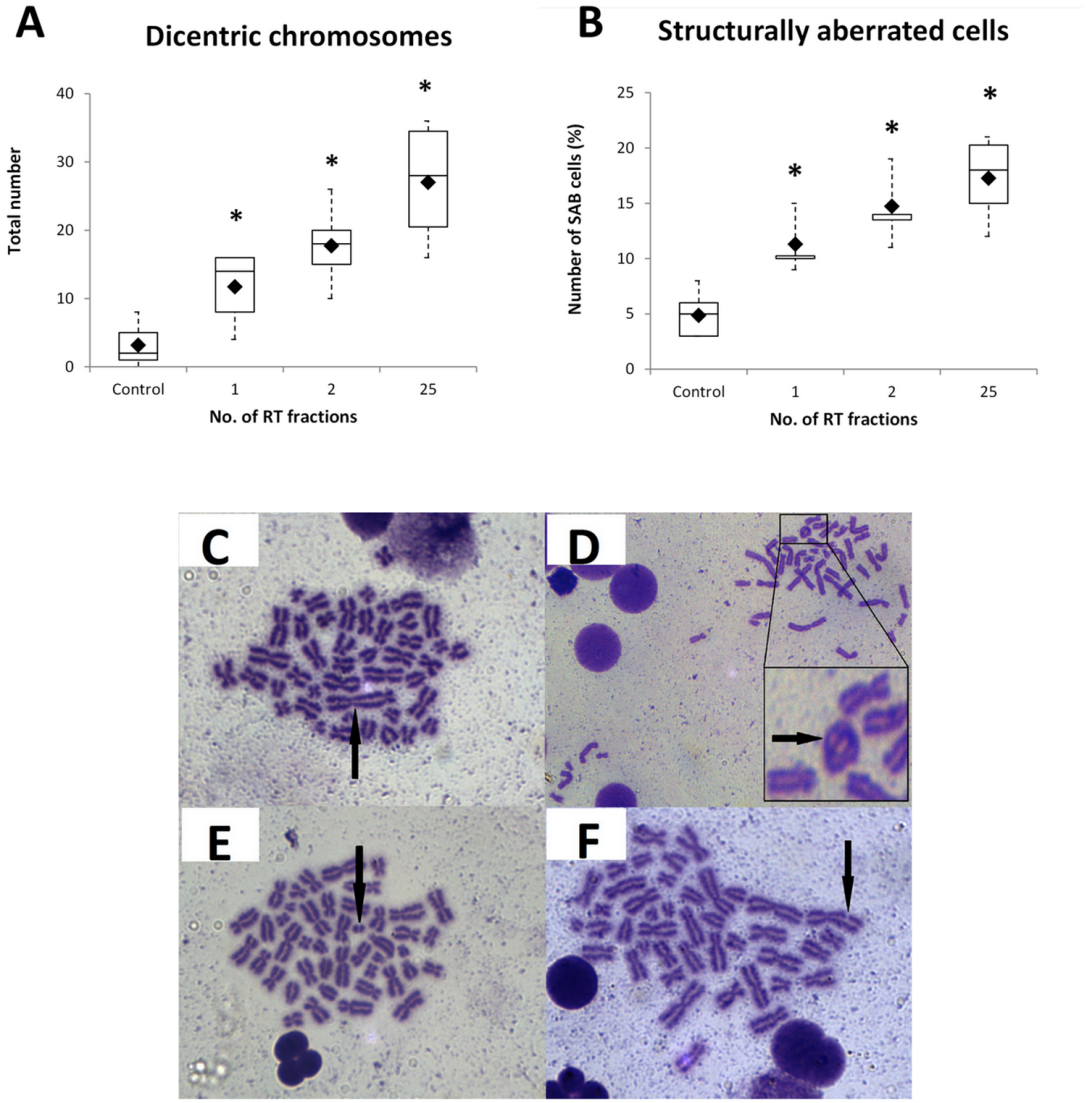
Chromosomal changes in PBMCs of head and neck patients. Number of dicentric chromosomes (A) and structurally aberrant cells (B) was determined and both parameters increase with the number of RT fractions. *, statistically significant difference versus Control group (p < 0.01). Representative figures of dicentric chromosome (C) and structurally aberant cells—ring chromosome (D), double fragment (E) and break (F) are shown.

We determined the number of DC per 100 cells and percentage of structurally aberrant cells in head and neck patients. We found the number of dicentrics at time 0, and after 1^st^, 2^nd^, and 25^th^ RT fractions, to be 1.5, 7.0, 9.0, and 12.5, respectively. The proportion of chromosomal aberrant cells (SAC) was 6, 11, 15, and 18%, respectively (Fig 2). The increase in the number of DC and SAC was significant in all groups when compared to the control group (p < 0.01).

### The number of micronuclei in PBMCs of endometrial patients increased with number of RT fractions

**Fig 3:**
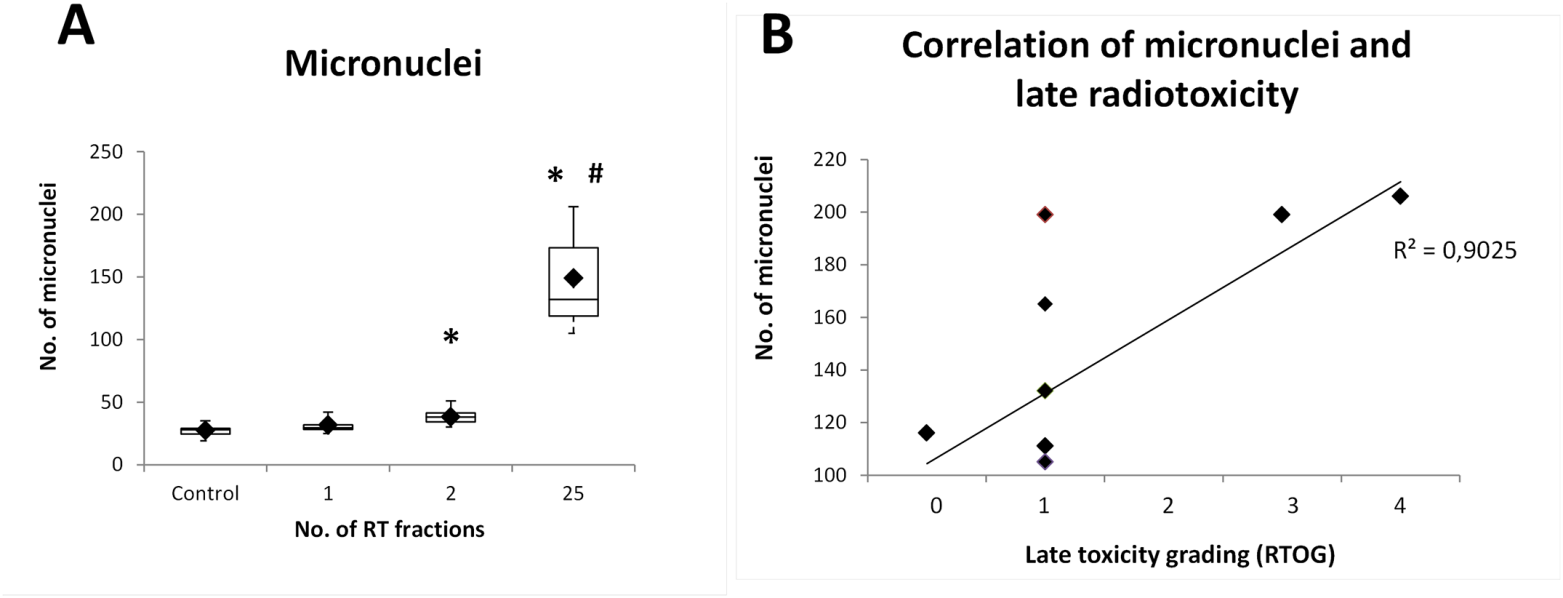
Incidence of micronuclei in PBMCs of endometrial patients (A) and their relation to late radiotoxicity (B). Number of MN increased with the number of RT fractions. BN, binucleated cells; *, statistically significant difference of the 2^nd^ and 25^th^ RT fraction versus Control group (p < 0.001); #, statistically significant difference of the 25^th^ RT fraction versus 2^nd^ RT fraction (p < 0.001).

We assessed the number of MN in ten endometrial patients at time 0 (27,40 ± SEM 4,45) and after 1^st^ (31.60 ± 4.74), 2^nd^ (38.10 ± 6.01), and 25^th^ (149.10 ± 37.33) RT fractions, respectively (Fig 3A). We observed statistically significant increase in the number of MN after the 2^nd^ and 25^th^ fraction when compared to the control group (p < 0.001). The increase after the 25^th^ RT fraction was also statistically significant when compared with the 2^nd^ RT fraction group (p < 0.001).

### Micronuclei could have potential for late radiotoxicity prediction

We evaluated late toxicity grading as the worst grade of symptoms persisting more than 3 months after the end of the treatment as described in Manning et al. [19]. Here we correlated our data with incidence of MN (Fig 3B). Eight out of ten patients exhibited late radiotoxicity graded 0 or 1. However, patients E7 and E9 suffered from late radiotoxicity effects (grade 4 and 3, respectively) and the highest number of MN we detected (206 and 199, respectively) corresponded with these patients. Although a larger group of patients would be required in order to confirm this conclusion, our results suggest that late radiation toxicity in endometrial patients might be associated with increased number of MN (correlation coefficient r^2^ = 0.902).

### The ratio of damaged cells in lymphocytes, PBMC, and granulocytes populations of both endometrial and head and neck patients did not changed throughout the whole study period

**Fig 4:**
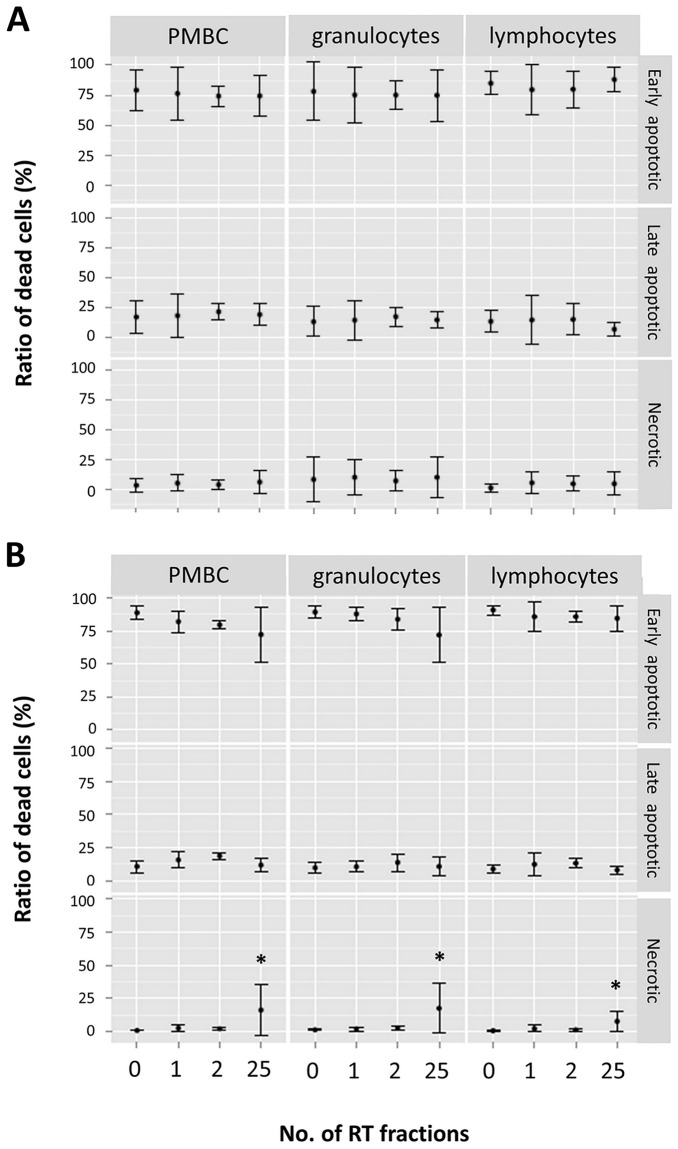
The relative proportion of early apoptotic, late apoptotic, and necrotic cells. The relative proportions of early apoptotic (Annexin V-positive/propidium iodide-negative), late apoptotic (Annexin V-positive/propidium iodide-positive), and necrotic cells (Annexin V-negative/propidium iodide-positive) were determined (all combined set as 100%) in PBMC, granulocytes, and lymphocytes populations of endometrial (A) and head and neck patients (B). None of them showed a statistically significant change, but the necrotic population in head and neck patients after 25 RT fractions. See S1 Fig for gating strategy. *, statistically significant difference of the 25^th^ RT fraction versus Control group (p < 0.05).

We measured the percentage of damaged cells, i.e. early apoptotic cells (Annexin V-positive/propidium iodide-negative), late apoptotic (Annexin V-positive/propidium iodide-positive), and necrotic (Annexin V-negative/propidium iodide-positive in lymphocytes, PBMC, and granulocytes population, respectively, by flow cytometry and observed no statistically significant change in both groups of endometrial and head and neck patients throughout all time intervals (Fig 4A and 4B). Interestingly, the proportion of necrotic cells in head and neck patients increased significantly at the end of therapy (after 25^th^ RT fraction) in all sub-populations when compared to the Control group (p < 0.05).

### The amount of mtDNA content decreased by time after irradiation

**Fig 5:**
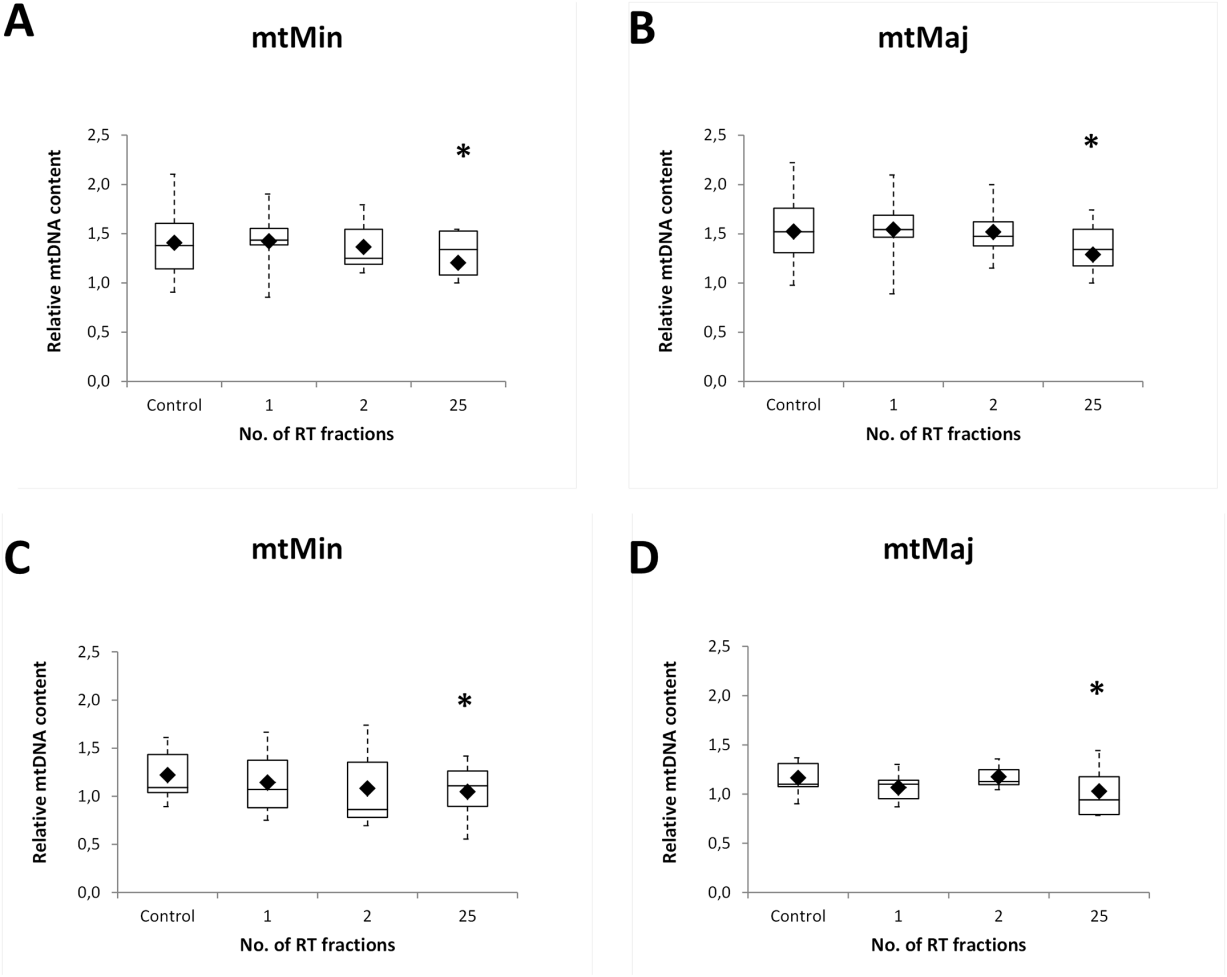
The amount of mtDNA content. mtDNA was quantified in the minor arc (mtMin) and in the major arc (mtMaj) site. The data indicate the relative decrease in mtDNA content over the time in both groups of the endometrial (A) and (B) and head and neck (C) and (D) patients. Upper and lower quartiles with median are shown. Black rhombs represent arithmetic mean; *, statistically significant difference between 25^th^ RT fraction and Control group (p < 0.05).

The level of reference gene B2M (measured DNA concentration in ng) was not changed during the studied time-points and we observed no time-dependent changes in mtDNA deletions. On the other hand, we observed statistically significant difference (p ≤ 0.05) of relative mtDNA content (mtMinArchand mtMajArch ratio) between control and 25^th^ RT fraction. Our data indicate that the relative mtDNA content decreased over the time in both groups of the endometrial (Fig 5A and 5B) and head and neck patients (Fig 5C and 5D) after 25^th^ RT fraction versus control time-point.

### Transcriptional modification of expression in vivo of radiation-responsive genes

Previously reported radiation responsive genes PHPT1, CCNG1, CDKN1A, GADD45, and SESN1 [4,20] were investigated in blood samples from RT patients to validate them *in vivo* for use in biodosimetry assays at early time-points (24 and 48 h) following one and two dose exposures, respectively. We also monitored the expression of these genes after the final RT fraction (5 weeks after the start of the RT treatment).

**Fig 6:**
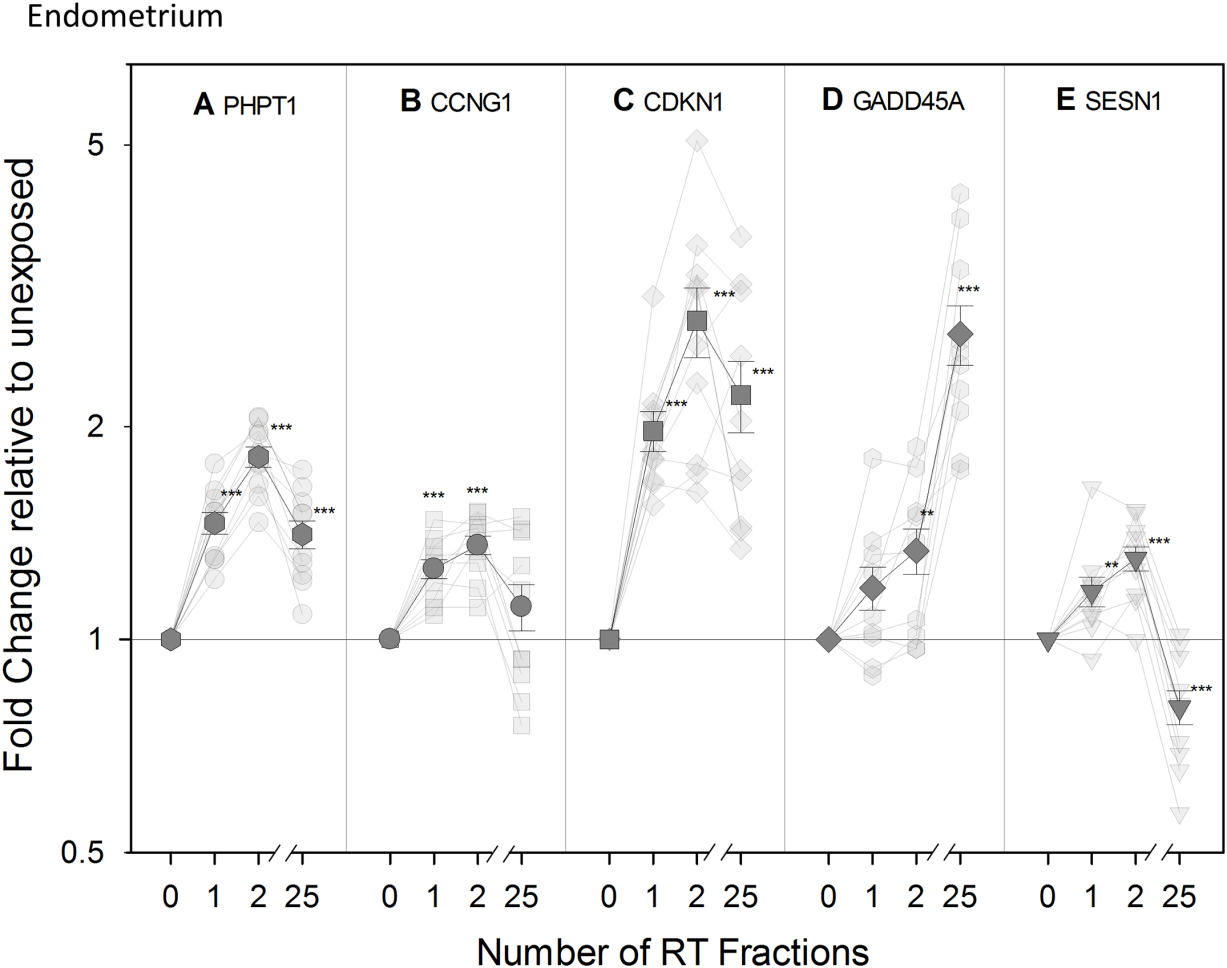
Transcriptional modification of expression of radiation-responsive genes in endometrial patients. Transparent symbols represent QRT-PCR fold change in expression of the genes PHPT (A), CCNG1 (B), CDKN1A (C), GADD45 (D), and SESN1 (E) for ten individual endometrium cancer patients before (0) and after 1, 2, and 25 fractions of radiotherapy (RT). The mean (± SEM) is shown as non-transparent symbols. Gene expression is given as fold change relative to unexposed Control sample set at 1 (normalized against the housekeeping gene, HPRT). **, statistically significant difference versus Control group (p < 0.01); ***, statistically significant difference versus Control group (p < 0.005).

**Fig 7:**
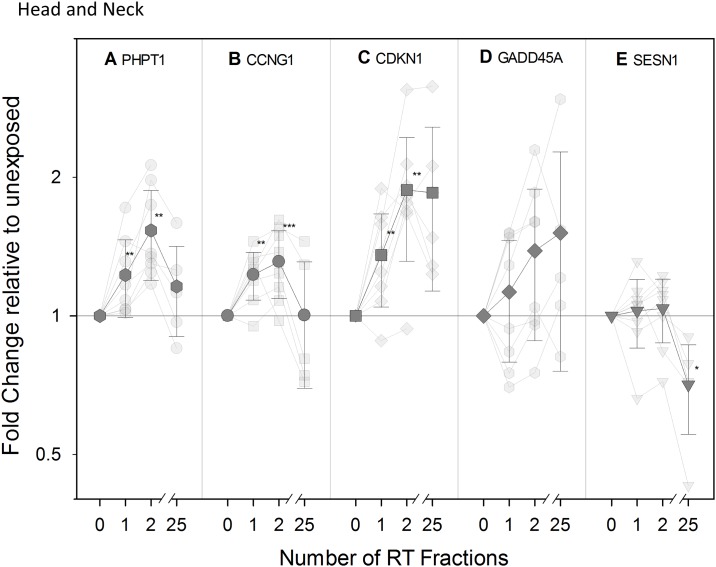
Transcriptional modification of expression of radiation-responsive genes in head and neck patients. Transparent symbols represent QRT-PCR fold change in expression of the genes PHPT (A), CCNG1 (B), CDKN1A (C), GADD45 (D), and SESN1 (E) for eight head and neck cancer patients before (0) and after 1, 2, and 25 fractions of radiotherapy (RT). The mean (± SEM) is shown as non-transparent symbols. Gene expression is given as fold change relative to unexposed Control sample set at 1 (normalized against the housekeeping gene, HPRT). **, statistically significant difference versus Control group (p < 0.01); ***, statistically significant difference versus Control group (p < 0.005). For GADD45 p-trend was applied (r^2^ = 0.980).

Overall, an up-regulation of PHPT1, CCNG1, CDKN1A, GADD45, and SESN1 was found in endometrial (Fig 6) and head and neck cancer patients (Fig 7) at all time points with the exception of SESN1. It showed a significant up-regulation at 48 h followed by a down-regulation becoming significant for endometrial patients following the 25^th^ RT fraction (Fig 6E). For many of the genes, the transcriptional response was more pronounced in endometrial cancer patients, who received a higher fractional dose to the blood. Hence, PHPT1 and CDKN1A were significantly up-regulated at all time points in endometrial patients (Fig 6A and 6C, respectively) while a significant up-regulation in head and neck cancer patients was observed for PHPT1 and CDKN1A only at 48 hr, after the 2^nd^ RT fraction (Fig 7A and 7C, respectively). CCNG1 (Cyclin G1) showed an up-regulation significant in the endometrial cancer patient group (Fig 6B) at the early time-points of 24 hr and 48 hr while in the head and neck cancer patients we detected up-regulations that were not statistically significant (Fig 7B). These genes therefore seem particularly suited for biodosimetry with an up-regulation 24 hr following a partial body exposure. GADD45 behaved slightly differently as the up-regulation observed was significant at the latest time-point specifically and only in the endometrial group (Fig 6D) although a similar trend could be observed in the head and neck group (Fig 7D) with r^2^ = 0.980. The gene SESN1, Sestrin 1 (Figs 6E and 7E), demonstrated a clearly different pattern of expression as the up-regulation seen at 48 hr was followed by a down-regulation found significant in the endometrial group following the 25th RT fraction (Fig 6E); a similar trend could be observed in the head and neck group.

## Discussion

In case of a large-scale radiological event, the subsequent medical management would strongly rely on triage of potentially exposed individuals. This can be achieved by assessing clinical signs and/or symptoms indicating severe exposure [21], however, procedures for determination of low doses are not fully developed and current biodosimetry suffers from certain gaps in the methodology [22] in the terms of sensitivity, limited high-throughput capacity etc. In this study, we wanted to compare different biomarkers of radiation exposure. To this end, we performed *in vivo* analysis of cytogenetic parameters, gene expression and mtDNA in oncological patients undergoing PBI.

So far, the “gold standard” of biological dosimetry is the dicentric chromosomal aberration assay. DC, rings, and fragments are generally considered as radiation-specific and these types of aberrations are referred to as unstable because their persistence in the body declines with cell division cycles [23]. *In vivo*, Matsuoka *et al*. detected chromosomal aberrations in lymphocytes from patients with tumors of stomach, prostate, lung, or hepatocellular carcinoma, who received high doses of therapeutic X-rays [24]. Many other studies have confirmed cytogenetic assays as valid tools for application in biodosimetry [25–27] and later, the high throughput capacity of this assay for triage purposes in the web-based scoring mode was suggested [3].

In this study, due to time and staff limitations we had to perform cytogenetic analysis in different laboratories, while each of them relied on different methodology (i.e. DC versus MN). Therefore, we analysed DC only in head and neck and MN in endometrial patients.

As expected, we found the number of DC as well as the number of chromosomal aberrations increasing in all head and neck tumor patients after the 1^st^ and the 2^nd^ RT fraction. The significant increase was observed also after 5 weeks, i.e. in the end of the therapy (25 RT fractions). Similar study was performed by Roch-Lefèvre *et al*. with eight patients treated for head and neck cancer, who observed a significant increase in the cytogenetic markers post-irradiation [28].

MN are formed during the metaphase/anaphase transition of mitosis and its scoring can be performed relatively easily and on different human cell types relevant for biomonitoring. Regarding the workload, this assay is much faster than DC analysis. The number of MN in PBMCs of endometrial patients increased significantly after each RT fraction that we examined. Similarly, Silva-Barbosa et al. assessed five uterine cancers patients irradiated by the absorbed dose of 0 Gy, 0.08 Gy, and 1.8 Gy, respectively, and found statistical increase in DC and MN, respectively [29]. A significant work has been conducted in the area of MN analysis and high-throughput biodosimetry by Dr. Brenner’s group [30,31], who recently reported an implementation of commercial robotic high-throughput screening system for large-scale radiological incidents [32].

In summary, based on results from cytogenetic analysis we conclude that the doses applied to PBI patients were high enough to produce a response on cytogenetic level. Unfortunately, our efforts to provide dose estimates from DC data were not successful due to large uncertainties including inter-individual variance and overdispersion, thus it was not possible to form any conclusions here.

In the terms of apoptosis induction, we aimed to measure damaged (not healthy) cells by flow cytometry and determined the relative proportion of early apoptotic, late apoptotic, and necrotic cells in lymphocytes, PBMC, and granulocytes population, respectively, and observed no statistically significant changes in both groups of patients throughout all time intervals. This was probably due to elimination of damaged cells by mononuclear phagocyte system. The immediate clearance of apoptotic cells by macrophages is crucial to inhibit inflammation and autoimmune responses and possibly did not allow us to monitor any changes [33].

In this study, novel quantitative biodosimetric assays were compared using *in vivo* blood samples of RT-treated patients against the golden standard methods. This study was performed as a one-year pilot and the low number of patients is a drawback. Nevertheless, such experiment is unique as to our best knowledge only one group reported assessment of mtDNA content as a marker of irradiation in humans *in vivo*. Wen et al. screened gene expression profiles of peripheral lymphocytes [34], but involved TBI patients irradiated with fairly high doses. The samples were taken 24 h after two RT sessions of 4.5 Gy over two successive days (9 Gy in total) from four adult acute lymphoid leukaemia patients. They observed 478 significantly expressed genes and identified three unique patterns. Furthermore, Wen *et al*. studied mtDNA content and common deletion levels in another set of 26 TBI patients by quantitative PCR. They reported significant modifications of both parameters and proposed them as predictive factors to radiation toxicity [12]. However, in our conditions we did not find any significant change in mtDNA deletion, possibly because we used PBI patients with lower mean blood doses. Nevertheless, we observed a time-dependent decrease in total mtDNA content in both groups of the PBI patients consistent with a detrimental radiation effect on lymphocytes mitochondria.

We and others have previously reported several radiation-responsive candidate genes potentially suitable for biological dosimetry purposes in several publications [4–7,35,36], but more has to be learnt about their applicability under *in vivo* conditions when applied to differentially exposed individuals. For example, *FDXR* is a well-known radiation responsive gene that has been formerly investigated for use in biodosimetry [7] but data obtained in regards to this gene will be presented elsewhere. Thus, here we aimed to assess and potentially validate *in vivo* the following genes previously identified after *ex vivo* exposure: *PHPT1*, *CCNG1*, *CDKN1A*, *GADD45*, and *SESN1*.

Overall, the genes that we studied *in vivo* in RT patients followed the same pattern of transcriptional expression in both groups of PBI patients, but the transcriptional response was more pronounced in endometrial cancer patients, compared to the head and neck patients. One possible explanation is that the irradiated volume is smaller in head and neck patients (i.e. in average 1.1 ± 0.17 versus 0.5 ± 0.11 dm^3^) and this is consistent with a lower dose to the blood and the lower doses delivered (i.e. mean dose per fraction 0.188 ± 0.20 versus 0.092 ± 0.012 Gy). We conclude that *CDKN1A*, *CCNG1*, and *PHPT1* are particularly suitable for biodosimetry with an up-regulation at early time-points.

Amongst the five genes analysed, four of them, *CCNG1*, *CDKN1A*, *GADD45* and *SESN1* are known to be transcriptionally regulated (at least partially) by the tumor suppressor protein p53. Expression of *CDKN1A*, also known as cell cycle inhibitor p21, is controlled by p53 in response to multiple stress stimuli such as DNA damage following radiation exposure. It is tempting to suggest that its persistent up-regulation at the transcriptional level might be due to genomic instability in cell mitochondria as shown by the decrease in total mtDNA content we reported although no mitochondrial deletions were found. *CDKN1A* up-regulation is significantly maintained throughout the treatment in both sets of patients and, although this was following 25 fractions of radiotherapy, it could potentially be useful for follow-up/retrospective biodosimetry. However, the expression of *CDKN1A* has also been associated with normal tissue sensitivity to RT [21] and its response has been variable in *ex vivo* irradiated blood samples [37].

Similarly to *CDKN1A*, *GADD45* (coding for Growth arrest and DNA damage-inducible 45 protein) expression level is clearly up-regulated after the 25^th^ fraction, while perhaps surprisingly *SESN1* is down-regulated significantly in endometrial patient blood (with a similar pattern in head and neck patients) at this final time-point. *SESN1* codes for sestrin 1, which plays a role in the cellular response to DNA damage and the oxidative stress response; the down-regulation towards the end of the treatment perhaps might be related to the cumulative doses and a chronic oxidative stress.

In addition, the gene PHPT1 (phosphohistidine phosphatase 1, the only one known in mammals) is, alongside *CDKN1A*, consistently up-regulated in both sets of patients. This gene regulates the phosphohistidine levels of several proteins including those involved in cell signalling, lipid metabolism, and potassium ion transport [38]. Perhaps it is therefore not surprising that it is up-regulated in lymphocytes following radiation exposure. Our results suggest that it is a strong candidate for biological dosimetry purposes.

*CCNG1* codes for cyclin G1 regulating cyclin-dependent protein kinases, which control cell cycle. We found *CCNG1* up-regulated only in endometrial patients. Up-regulation was not significant towards the end of RT, making it suitable for biodosimetry of single exposures.

Importantly, we performed dose estimates on the gene expression data generated; however, they were quite variable amongst patients. We did not observe any increase in dose estimate with increasing dose to the blood and the dose estimates were sometimes high in the pre-exposure controls. The best gene for dose estimates was *GADD45* but even then it did not exhibit a significant correlation between the dose and its expression. Although the data were not included here, a comparison with other radiation-responsive genes would be needed to identify the best biomarkers of exposure in *in vivo* samples. So far, we suggest that the best candidate gene for biological dosimetry proposed by our group would still be *FDXR* as reported previously [7,36,39] due to high linear-dynamic range and low inter-individual variance.

It should be noted that the role of potential confounding factors such as age and gender (in case of head and neck patients) could not be evaluated due to limited sample size but would be of importance for the future studies. Several studies suggested the use of different biomarkers as potential tools for normal tissue response prediction. As the cumulative doses used in this study reach high levels, tools such as this could be used to monitor radiotherapy responses as well. Although our data would require confirmation and extension, it indicate that late radiation toxicity in RT patients might be associated with MN increase, which would support the use of MN as cytogenetic predictive markers for clinical radiosensitivity and underlie a prognostic role of such biological parameter for patients’ follow-up. It should be noted that we evaluated response to fractionated doses. Thus, the translation of our findings to a single dose scenario is limited but still it is the only feasible way of conducting such study in humans.

**Fig 8:**
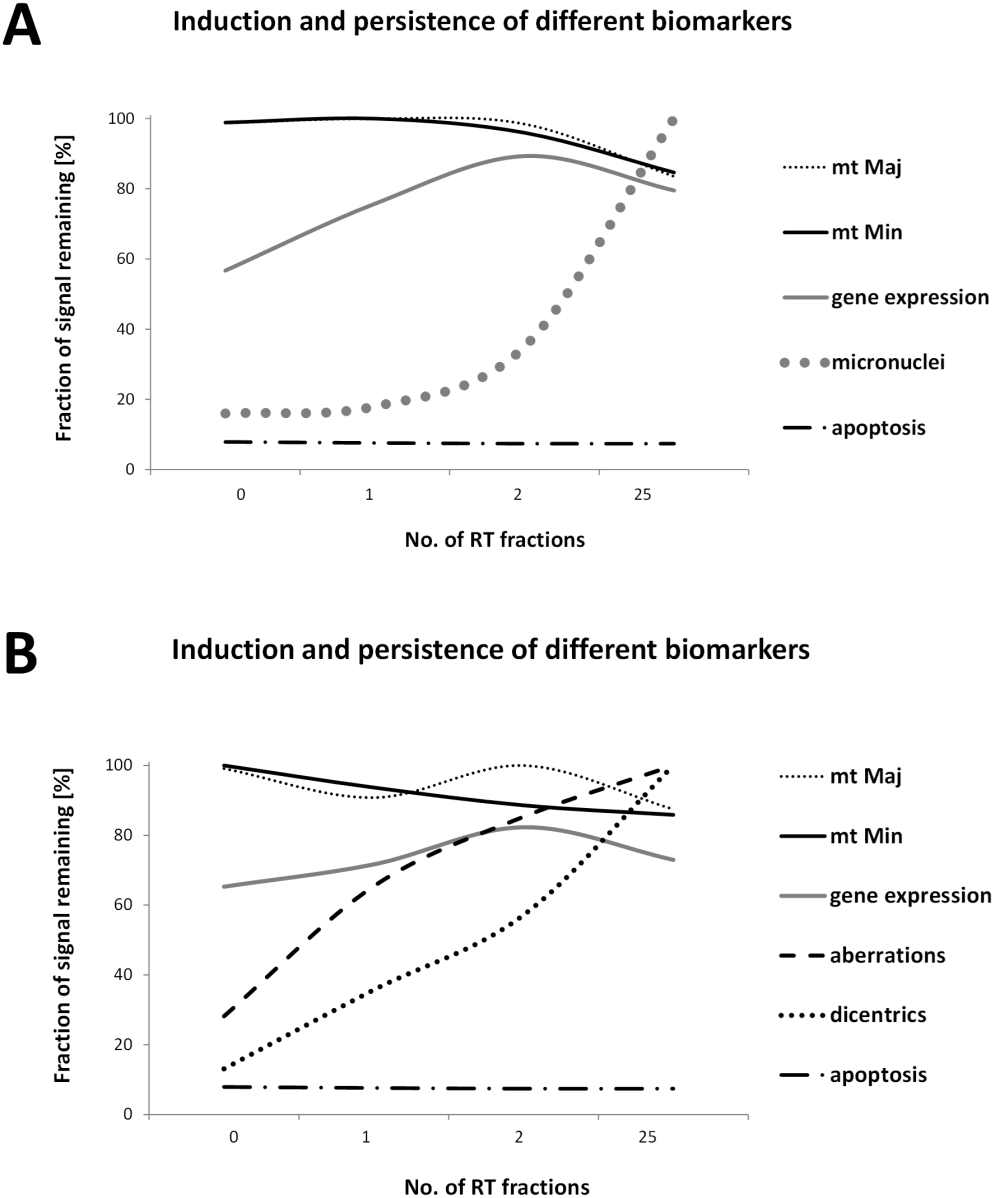
Induction and persistence of different biomarkers. Graphs showing the induction and temporal persistence of the biomarkers studied in endometrial (A) and head and neck patients (B). In order to compare all the parameters studied, the highest value detected was arbitrary set as 100%. Established cytogenetic markers and mtDNA content are suitable for late time points after irradiation while the new biomarkers as radiation-induced genes are more informative atearly time-points after radiation exposure. mt Maj, data from mtDNA content analysis of mtMajArch; mt Min, data from mtDNA content analysis of mtMinArch; gene expression, data from MQRT-PCR (average of five genes); micronuclei, data from micronuclei assay; aberrations, data from structural aberration analyses; dicentrics, data from dicentric chromosomes assay; apoptosis, data from flow-cytometric detection of apoptotic cells in lymphocyte population (percentage of apoptotic cells versus healthy-intact cells set as 100%).

Above that, this is the first attempt to compare several cellular and molecular radiation biomarkers in human peripheral blood irradiated *in vivo* in the same patients in a single study. Combining emerging and established biomarkers has the potential to provide more accurate monitoring of individual response to IR exposure and possibly, to contribute to assessment of the radiation toxicity and long term effects. Based on our data, we constructed graphs showing the induction and temporal persistence of the biomarkers studied here (Fig 8). Zeegers et al. compared suitability of different current biodosimetric markers. Although they performed their study on a cell line and not *in vivo* they conclusions strongly encouraged the idea of employing a multiparametric approach for dose/risk estimation [40].

Overall, in the case of PBI and multiple fractions it appears that early-time points are better monitored by radiation induced genes than the later time-points where the use of cytogenetic markers might be more beneficial.

## Conclusion

In summary, the results discussed here represent the first study of its kind that used *in vivo* model of PBI oncological patients undergoing RT to compare several divergent cellular and molecular biomarkers of radiation exposure.

Cytogenetic data confirmed the expected dose-dependent increase in DC and induction of MN in PBMCs. Similarly, increasing the number of RT fractions led to a decrease in mtDNA content. Thus, we suggest that although we were not able to confirm mtDNA content to have a potential as biomarker of a single radiation exposure, we suggest that more work should be carried out to confirm that it could be of importance in situations following multiple fraction doses and that its decrease might have physiological consequences. Finally, monitoring the transcription of five genes previously identified in *ex vivo* experiments as radiation responsive, highlighted the sensitivity and consistency across individuals of these biomarkers.

It seems that established cytogenetic markers (DC and MN) and mitochondrial DNA content are suitable for later time points after irradiation while the new biomarkers as radiation-induced genes are suitable for rapid monitoring of early time-points after radiation exposure.

Importantly, growing evidence indicates the upcoming trend in research of indicators of ionising radiation effects. It will involve a combination of established and emerging biomarkers and our study provides a platform for future work in this area. Among these biomarkers, some have the potential for use in radiation oncology as they can be easily monitored during the course of the RT treatment.

## Supporting information

S1 Fig Gating strategy for flow-cytometry (Annexin V) experiment. (A) Dot plot analysis shows the region of lymphocytes (R1), PBMC (R2) and granulocytes (R3) based on their forward scatter (FSC) and side scatter (SSC) characteristic. (B) Dot plot analysis of lymphocytes (R1 region) shows intact cells (Annexin V-negative, PI-negative), early apoptotic cells (Annexin V-positive, PI-negative), apoptotic cells (Annexin V-positive and PI-positive) and necrotic cells (Annexin V-negative, PI-positive) in the peripheral blood of PBI patient. (C) Dot plot analysis of PBMC (R2 region) shows intact cells (Annexin V-negative, PI-negative), early apoptotic cells (Annexin V-positive, PI-negative), apoptotic cells (Annexin V-positive and PI-positive) and necrotic cells (Annexin V-negative, PI-positive) in the peripheral blood of PBI patient. (D) Dot plot analysis of granulocytes (R3 region) shows intact cells (Annexin V-negative, PI-negative), early apoptotic cells (Annexin V-positive, PI-negative), apoptotic cells (Annexin V-positive and PI-positive) and necrotic cells (Annexin V-negative, PI-positive) in the peripheral blood of PBI patient.
(TIF)
