# Supplementary figures and images for: The first in vivo multiparametric comparison of different radiation exposure biomarkers in human blood
Source: PLoS One. 2018 Feb 23;13(2):e0193412. doi: 10.1371/journal.pone.0193412 (PMC5825084; doi:10.1371/journal.pone.0193412)

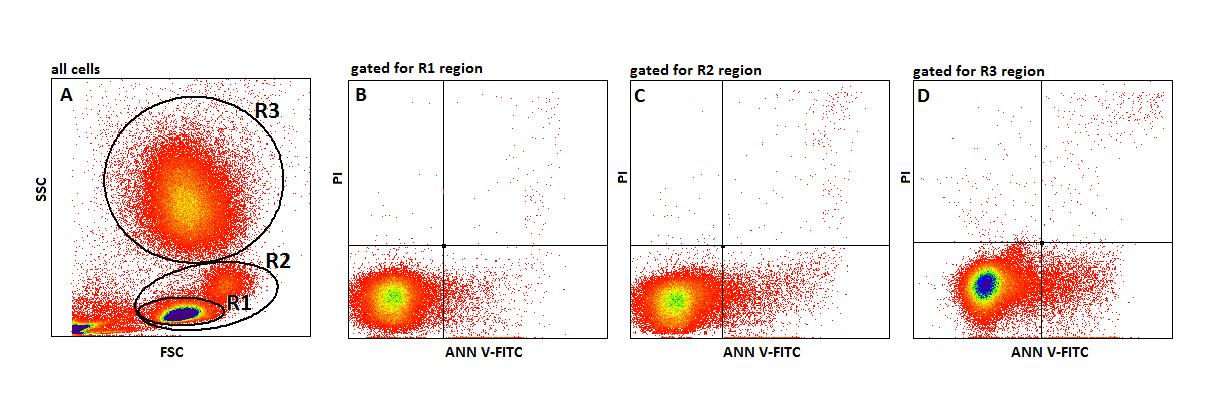

Supplement: S1 Fig — (A) Dot plot analysis shows the region of lymphocytes (R1), PBMC (R2) and granulocytes (R3) based on their forward scatter (FSC) and side scatter (SSC) characteristic. (B) Dot plot analysis of lymphocytes (R1 region) shows intact cells (Annexin V-negative, PI-negative), early apoptotic cells (Annexin V-positive, PI-negative), apoptotic cells (Annexin V-positive and PI-positive) and necrotic cells (Annexin V-negative, PI-positive) in the peripheral blood of PBI patient. (C) Dot plot analysis of PBMC (R2 region) shows intact cells (Annexin V-negative, PI-negative), early apoptotic cells (Annexin V-positive, PI-negative), apoptotic cells (Annexin V-positive and PI-positive) and necrotic cells (Annexin V-negative, PI-positive) in the peripheral blood of PBI patient. (D) Dot plot analysis of granulocytes (R3 region) shows intact cells (Annexin V-negative, PI-negative), early apoptotic cells (Annexin V-positive, PI-negative), apoptotic cells (Annexin V-positive and PI-positive) and necrotic cells (Annexin V-negative, PI-positive) in the peripheral blood of PBI patient. (TIF) [file pone.0193412.s001.tif]
